# The complete chloroplast genome sequence of herb *Nardostachys jatamans* (family: Valerianaceae) in China

**DOI:** 10.1080/23802359.2020.1861568

**Published:** 2021-01-27

**Authors:** Jiabin Deng, Heng Liang, Xuqiang Luo, Wei Zhang, Gang Gao, Ruiwu Yang, Khawaja Shafique Ahmad, Guiling Zhang

**Affiliations:** a School of Geography and Resources, Guizhou Education University, Guiyang, China; b College of Life Science, Sichuan Agricultural University, Yaan, China; c College of Life Sciences and Food Engineering, Yibin University, Yibin, China; d Department of Botany, University of Poonch Rawalakot, Azad Jammu and Kashmir, Pakistan; e School of Chemistry and Materials Science, Guizhou Education University, Guiyang, China

**Keywords:** *Nardostachys jatamans*, chloroplast genome, phylogenetic analysis

## Abstract

*Nardostachys jatamans* is an endemic herb in China, distributes mainly in Southeast Gansu, South Qinghai and West Sichuan of Qinghai-Tibet Plateau. In this study, the complete chloroplast genome (a typical quadripartite structure) sequence of *N. jatamans* was reported. The length of the DNA molecule was 155,268 bp with a large single-copy region (LSC: 87,263 bp), small single-copy region (SSC: 17,327 bp) and inverted repeats (IRa and IRb: 25,339 bp). The overall GC content was 38.56%. It has a total of 129 genes, containing 83 protein-coding genes, 38 tRNA genes, and eight rRNA genes. The phylogenetic analysis has shown that *N. jatamans* is sister to *Valeriana offcinalis.* The chloroplast genome provides the basis for development and utilization of *N. jatamans* in future.

*Nardostachys jatamans,* locates in Southeast Gansu, South Qinghai and West Sichuan of Qinghai-Tibet Plateau in China, contains multiple chemical compositions including alkaloids, minor triterpenes, steroids, lignans, flavonoids, coumarins, lignan and terpenoids, etc. (Paek and Lim 2014), is an endemic traditional medicinal herb for its biological activities (anti-depressant, anti-arrhythmia, anti-oxidation, anti-tumor and anti-inflammation, etc.) (Wu et al. 2011; Liu et al. 2013; Zhang et al. 2015; Wu et al. 2018).

Fresh leaves of *N. jatamans* was collected from the Autonomous Prefecture of Aba Tibet and Qiang Race of Sichuan Province and stored in the refrigerator at −80 °C. Voucher specimen (No. GS-AB-01) was deposited in the herbarium of Guizhou Education University. Total genomic DNA was isolated by using a modified CTAB method (Doyle 1987). The library with insert size of 300 bp fragments was sequenced by Illumina Hiseq 2500 Sequencing System (Illumina, Hayward, CA, USA) in Genepioneer Biotechnologies Co. Ltd, Nanjing, China and the lib The NOVOPlasty software was used to assemble the raw reads (Dierckxsens et al. 2017). CLC Genomics Workbench v8.0 was used to filter out the low-quality sequences and the chloroplast genome was reconstructed by MITObim v1.8 (Hahn et al. 2013). The complete chloroplast genome of *N. jatamans* was annotated in Geneious Prime (Kearse et al. 2012) and aligning with relatively related species. The data of complete chloroplast genome sequence of *N. jatamans* were submitted to GenBank (MW149527). The complete cp genome of *N. jatamans* was 155,268 bp in length. There was a circular DNA molecule with typical quadripartite structure (IRa and IRb: 87,263 bp, LSC: 87,263 bp and SSC: 17,327 bp). The overall GC content was 38.56%. The GC content was 36.77%, 33.3% and 43.42% in the regions of LSC, SSC and IR, respectively. GC content of IRs region is the highest. There were 22 genes have only one intron, and three genes have two introns. There were 129 genes, containing 83 protein-coding genes, 38 tRNA genes, and eight rRNA genes in the complete chloroplast genome sequence of *N. jatamans*.

To investigate the phylogeny of *N. jatamans*, 26 taxa were included (15 species from Caprifoliaceae, 5 species from Valerianaceae, 4 species from Dipsacaceae and 2 species as outgroups). All sequences were aligned in MAFFT (Katoh and Standley 2013) and manually adjusted by using trimAL version 1.4 (Capella-Gutiérrez et al. 2009). RAXML version 8 (Stamatakis 2006) was used to infer maximum likelihood (ML) tree chosen GTRGAMMA model with 1000 rapid bootstrap. The phylogenetic tree has shown that *N. jatamans* and *Valeriana offcinalis* were fallen into a branch with robust bootstrap support values in Valerianaceae (Figure 1).

**Figure 1.**
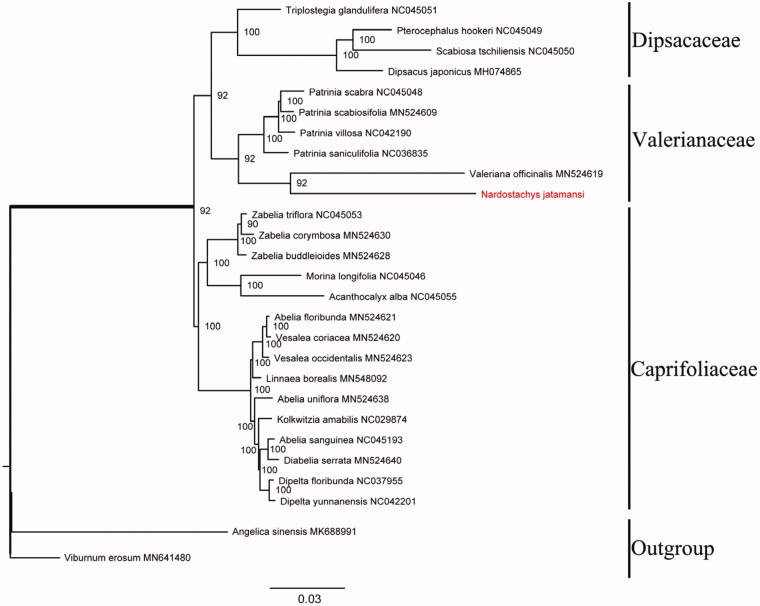
Maximum-likelihood phylogenetic tree based on 27 complete chloroplast genomes. The ML bootstrap support value is on each branch. The Chloroplast genomic accession numbers used in this phylogeny analysis: *Patrinia scabra* (NC045048), *Patrinia scabiosifolia* (MN524609), *Patrinia villosa* (NC042190), *Patrinia saniculifolia* (NC036835), *Valeriana officinalis* (MN524619), *Acanthocalyx alba* (NC045055), *Kolkwitzia amabilis* (NC029874), *Diabelia serrata* (MN524640), *Abelia floribunda* (MN524621), *Abelia sanguinea* (NC045193), *Abelia uniflora* (MN524638), *Pterocephalus hookeri* (NC045049), *Dipelta yunnanensis* (NC042201), *Morina longifolia* (NC045046), *Dipelta floribunda* (NC037955), *Vesalea occidentalis* (MN524623), *Vesalea coriacea* (MN524620), *Zabelia triflora* (NC045053), *Zabelia corymbosa* (MN524630), *Zabelia buddleioides* (MN524628), *Kolkwitzia amabilis* (NC029874), *Linnaea borealis* (MN548092), *Triplostegia glandulifera* (NC045051), *Dipsacus japonicus* (MH074865), *Scabiosa tschiliensis* (NC045050), *Viburnum erosum* (MN641480), and *Angelica sinensis* (MK688991).

## Data Availability

The data that support the findings of this study are openly available in GenBank of NCBI at https://www.ncbi.nlm.nih.gov, reference number MW149527.
